# The *BLADE-ON-PETIOLE* genes of Arabidopsis are essential for resistance induced by methyl jasmonate

**DOI:** 10.1186/1471-2229-12-199

**Published:** 2012-11-02

**Authors:** Juan Vicente Canet, Albor Dobón, Jana Fajmonová, Pablo Tornero

**Affiliations:** 1 Current address: Department of Crop Genetics, The John Innes Centre, Norwich Research Park, Norwich, NR4 7UH, UK; 2 Current address: Department of Economics and Management of Chemical and Food Industry, Faculty of Chemical Engineering, Institute of Chemical Technology Prague (ICT), Technická 5, Prague 6, Dejvice, 166 28, Czech Republic; 3 Instituto de Biología Molecular y Celular de Plantas (IBMCP), Universidad Politécnica de Valencia (UPV)-Consejo Superior de Investigaciones Científicas (CSIC), Ciudad Politécnica de la Innovación (CPI), Ed. 8E; C/ Ingeniero Fausto Elio s/n, Valencia, 46022, Spain

**Keywords:** Methyl jasmonate, Salicylic acid, Arabidopsis, NPR1, BOPs, Defense

## Abstract

**Background:**

*NPR1* is a gene of *Arabidopsis thaliana* required for the perception of salicylic acid. This perception triggers a defense response and negatively regulates the perception of jasmonates. Surprisingly, the application of methyl jasmonate also induces resistance, and *NPR1* is also suspected to be relevant. Since an allelic series of *npr1* was recently described, the behavior of these alleles was tested in response to methyl jasmonate.

**Results:**

The response to methyl jasmonate of different *npr1s* alleles and *NPR1* paralogs null mutants was measured by the growth of a pathogen. We have also tested the subcellular localization of some npr1s, along with the protein-protein interactions that can be measured in yeast. The localization of the protein in *npr1* alleles does not affect the response to methyl jasmonate. In fact, *NPR1* is not required. The genes that are required in a redundant fashion are the *BOPs*. The *BOPs* are paralogs of *NPR1*, and they physically interact with the TGA family of transcription factors.

**Conclusions:**

Some *npr1* alleles have a phenotype in this response likely because they are affecting the interaction between BOPs and TGAs, and these two families of proteins are responsible for the resistance induced by methyl jasmonate in wild type plants.

## Background

Plants are constantly defending themselves against pathogens by means of a wide array of mechanisms. Some of them are pre-existing (or non inducible) and others are induced in response to the pathogen attack. Salicylic acid (SA, reviewed by [1]) is a plant hormone which is crucial for the inducible response of *Arabidopsis thaliana* (Arabidopsis) to biotrophic pathogens like *Pseudomonas spp*[2]. When a pathogen is perceived, SA is produced and accumulated, producing a proper defense. This SA signaling occurs not only where the attack takes place, since defense is also enhanced in leaves different from the one inoculated. This is called Systemic Acquired Resistance (SAR, [3]). SA has an intricate crosstalk with other hormones, showing an overall negative crosstalk with auxins, ethylene (ET), and jasmonates (JA, crosstalk of hormones reviewed by [4]). In the case of JA, it has been shown that the active form *in planta* is JA-Ile (reviewed by [5]), while in the laboratory is used exogenously as Methyl Jasmonate (MeJA).

*NON-EXPRESSOR OF PATHOGENESIS-RELATED GENES1* (*NPR1*) is the main gene required for SA perception [6]. There are five paralogs of *NPR1* in Arabidopsis [7], *BLADE-ON-PETIOLE1* (*BOP1*) and *BOP2* have an important role in development [8], *NPR3* and *NPR4* have a role in defense [9], probably through SA perception [10], and no specific function for *NPR2* has been described, besides a secondary role in SA perception [11]. There are other genes that are relevant for signal transduction, like the family of *TGA* transcription factors whose products interact with NPR1 [12], but they are required in a redundant fashion.

*NPR1* has been described as having more than one role in defense, since it is also important in the Induced Systemic Resistance (ISR, [13]). ISR is defined as the resistance triggered at the leaves by a non pathogenic organism inoculated in the roots, and while SAR requires SA signaling, ISR requires MeJA and ET signaling. As with SA, exogenous applications of MeJA and ET trigger resistance in Arabidopsis towards some biotrophs, like *Pseudomonas*[14]. It has been proposed that *NPR1* is relevant for the resistance-inducing ability of MeJA ([13], hereafter abbreviated as RIM), although RIM it is not necessarily equivalent to ISR. While the role of *NPR1* in SA perception takes place in the nucleus [15], its function in RIM is not so clearly understood. It has been described a cytosolic function of NPR1 crucial in cross-talk between SA and JA signaling [16]. Furthermore, Arabidopsis transcriptome analysis upon pathogen infection has suggested that such cytosolic function is also involved in the modulation of JA-dependent defenses [17]. The *npr1-*3 mutant, which produces a truncated cytoplasmatically localized npr1 protein with no nuclear localization signal, has been reported to be affected only in SA-dependent gene expression, not in JA and ET dependent genes. In contrast, the *npr1-*1 mutant, which has a mutation in a key domain, is affected in the expression of SA, JA and ET-dependent genes [17]. More recent studies support such cytosolic NPR1 function as regulator of JA-dependent defense responses ([18-20]).

*BOP1* was first identified by its mutant phenotype of ectopic blades along the petioles, as well as some alterations in the flowers [8]. The first allele identified was a dominant negative, since T-DNA insertions in *bop1* did not reproduce the phenotypes of ectopic blades [21]. Once *BOP1* was identified as paralog to *NPR1*[22], it was shown that other paralog, *BOP2*, functions redundantly with *BOP1*[21]. The double mutant *bop1 bop2* reproduced all the developmental phenotypes of ectopic blades, but it was wild type when inoculated with *Pseudomonas*[21], and it is also wild type for SA perception [23].

Since a collection of *npr1* alleles has recently been available [11], we tested the hypothesis that the role of *NPR1* in RIM is cytosolic. In this work, we show that *NPR1* has no role in RIM in wild type conditions, since the genes responsible for RIM are *BOP1* and *BOP2*, with an important part being played by the *TGAs*. Therefore, two genes required for the normal development of the leaf, are also required for plant defense.

## Results

### Role of NPR1 in RIM

*NPR1* has been characterized as a result of observing the response to SA of the great number of alleles described for it [24]. *NPR1* has also been described as essential for RIM, but there are differences between alleles, since *npr1*-1 and *npr1*-3 have different RIM ([20,25]). *npr1*-1 and *npr1*-3 have other differences in phenotypes related to MeJA. Thus, the SA-JA antagonism is not present in *npr1*-1, but it is active in *npr1*-3 [16]. Other difference is the gene expression, whereas *npr1*-1 was affected in SA, JA, and ET dependent genes upon *Pto* inoculation, *npr1*-3 was only affected in SA dependent genes [17]. These different phenotypes have been attributed to the lack of nuclear localization in *npr1*-3, since the truncated cytosolic protein would be functional to modulated JA-dependent defense response [19].

**Figure 1:**
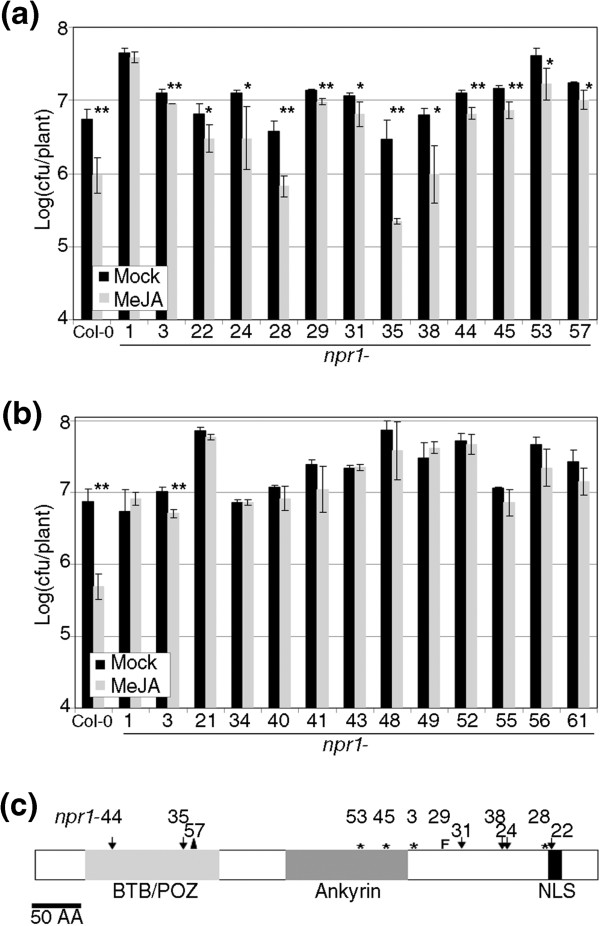
***npr1 *alleles differ in their response to MeJA.** Forty-three *npr1* alleles were treated with 100 μM methyl jasmonate (MeJA), with 0.1% DMSO and 0.02% Silwet L-77 or a mock treatment. One day later, *Pseudomonas syringae pv. tomato DC3000* (*Pto*) was inoculated and its growth measured three days later in a logarithmic scale. For each genotype and treatment three samples with 5 plants per sample were taken. The bars show the average ± SD and *npr1*-1 and *npr1*-3 are included as controls for negative and positive response to MeJA respectively. **(a) ***npr1* alleles that show resistance induced by MeJA. **(b) ***npr1* alleles that do not show resistance induced by MeJA. **(c)** Schematic representation of the *npr1* alleles that showed resistance induced by MeJA along the structure of NPR1. BTB/POZ stands for Broad-Complex, Tramtrack and Bric-a-brac proteins, Pox virus and Zinc finger proteins. Ankyrin for Ankyrin Repeat Motifs (4 of them) and NLS for Nuclear Localization Signal. The arrows indicate point mutations, the asterisks stop codons, the letter “F” frameshift, and a triangle an internal deletion. The number indicates the number of allele. In all figures, the experiments were repeated at least three times with similar results. One asterisk means a significant difference with P<0.05, and two asterisks means P<0.01 (Student’s test of one tail).

In order to determine the precise role of *NPR1* in RIM, and to asses the role of the cytosolic vs. nuclear localization, we tested an allelic series of 43 *npr1* alleles, defined by their inability to perceive SA [11]. The *npr1* alleles produced a mixture of phenotypes in RIM (Figure 1), and out of the 43 alleles tested, 11 had a significant RIM (RIM+, Figure 1a), meaning that the growth of *Pto* in MeJA treated plants was significantly lower than in mock treated plants (Student’s test of one tail, with P<0.05). The rest of alleles showed no response (RIM-, 11 out of 32 are shown in Figure 1b). These two categories of alleles did not share any obvious feature and had the same proportion of stops and point mutations in each category. In fact, in the RIM+ alleles tested, six point mutations were widely scattered along the protein (Figure 1c, *npr1*-3 is included as reference).

**Figure 2:**
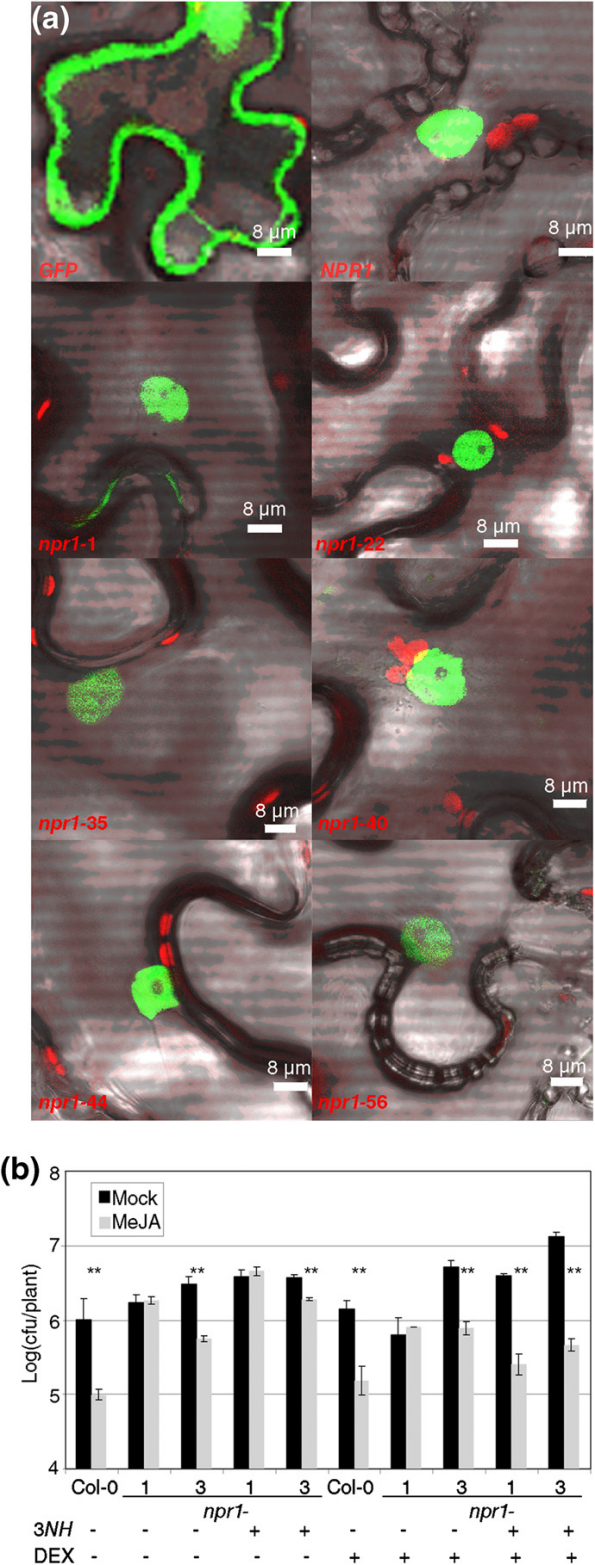
**The nuclear localization of *npr1 *alleles is not relevant for resistance induced by MeJA. (a)** Transient expression of *GFP*, *NPR1:GFP,* and six *npr1* alleles (three from Figure 1a and three from Figure 1b). *Agrobacterium tumefaciens* containing the mentioned genes under the promoter 35S were infiltrated in leaves of *Nicotiana benthamiana*, and the expression was detected with a confocal microscopy four days later. **(b)** The cytoplasmic anchoring of NPR1 does not complement *npr1-*1 in its response to MeJA. Plants with the transgene *35SCaMVp:NPR1:HBD* (abbreviated as 3*NH*) in *npr1*-1 or *npr1*-3 background and its controls were treated with either dexamethasone (DEX) or mock solution, and then treated with either MeJA or mock solution. One day later, *Pto* was inoculated and measured as described in Figure 1. One asterisk means a significant difference with P<0.05, and two asterisks means P<0.01 (Student’s test of one tail).

These alleles could somehow affect the localization of the protein inside the cell, even if the mutation was not in the NLS. To check this possibility, cDNAs of three RIM+ alleles (*npr1*-22, -35 and −44, Additional file 1), and three RIM- alleles (*npr1*-1, -40, and −56, Additional file 1), chosen among the point mutations, were cloned along with the wild type *NPR1*. Then, these seven cDNAs were transiently expressed in *N. benthamiana* under the control of the 35S promoter and with the GFP marker. The free GFP was detectable in the nucleus and in the cytoplasm. But when the wild type and the six alleles of *npr1* were expressed in the same conditions, GFP was detected mainly in the nucleus, with no difference existing between the two classes of alleles (Figure 2a).

As a complementary approach, we took advantage of the transgenic line that overexpresses NPR1 fused to the steroid hormone binding domain of the rat glucocorticoid receptor (HBD, and the transgenic plants are known as NPR1-HBD, [15]). NPR1-HBD remains exclusively in the cytosol in mock conditions and should be functional in RIM. The original line is in an *npr1*-3 background (RIM+), and therefore the transgene was transferred to an *npr1*-1 background (RIM-) to check for complementation. Treatments with BTH and with and without glucocorticoid dexamethasone (DEX) showed that NPR1-HBD was functional (Additional file 1). NPR1-HBD, even under the control of the 35S promoter, did not complement the lack of RIM in *npr1*-1 (Figure 2b). When DEX was applied, NPR1-HBD moved to the nucleus and *npr1*-1 was complemented in the RIM phenotype. Note that the presence of cytosolic NPR1-HBD in an *npr1*-3 background did not enhance RIM in comparison to *npr1*-3 alone.

**Figure 3:**
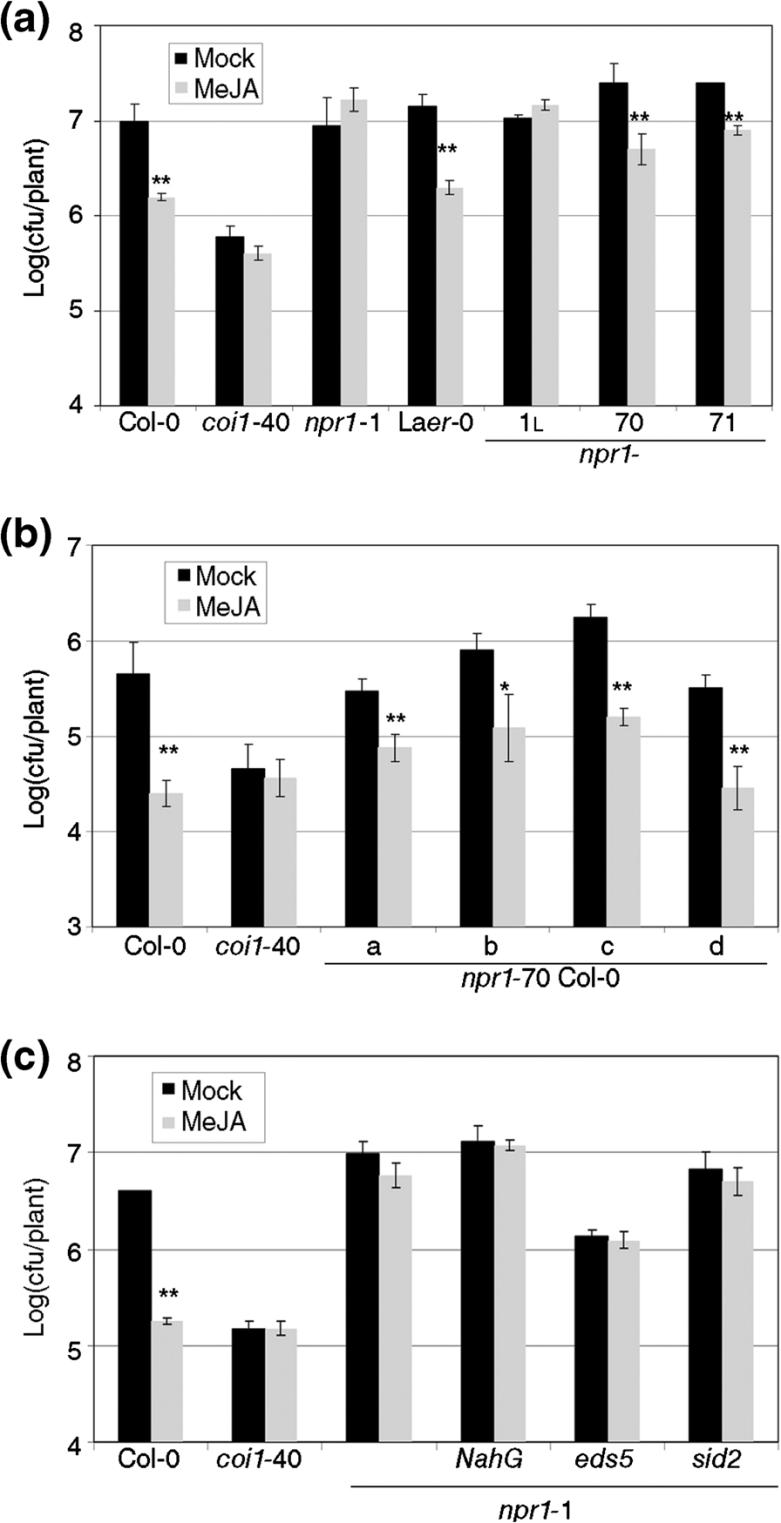
***NPR1 *is not necessary for resistance triggered by MeJA. (a)** Two null *npr1* alleles (*npr1*-70 and *npr1*-71, both in La*er*-0 background), plus their controls were tested for resistance induced by MeJA as described in Figure 1. *coi1*-40 is introduced as negative control for resistance triggered by MeJA. *npr1*-1_L_ is *npr1*-1 introgressed in Laer-0 [11]. **(b)** Introgressed lines of *npr1*-70 in Col-0 show the same phenotype than the original *npr1*-70. **(c)** The effect of some *npr1* alleles on resistance triggered by MeJA is not due to an excess of salicylic acid. Double mutants of *npr1*-1 with *NahG*, *eds5*, and *sid2* were tested for their response to MeJA in resistance. One asterisk means a significant difference with P<0.05, and two asterisks means P<0.01 (Student’s test of one tail).

The *npr1* alleles RIM+ or RIM- did not share any obvious feature, so it would be difficult to assign a precise role to the wild type gene. A critical genetic resource to discern the role of a gene is the null allele. Therefore, the response of two null alleles of *npr1* to RIM was measured (Figure 3a). Both *npr1*-70 and *npr1*-71 are in La*er*-0 background, so an introgression of *npr1*-1 in La*er*-0 was used as control [11]. These two null alleles responded to RIM in all experiments like the wild type. *npr1*-70 introgressed in Col-0 responded again like the wild type, which ruled out any ecotype effect (Figure 3b). Since the direct role of *NPR1* in RIM was in question as a consequence of the aforementioned results, we included an independent RIM- control, *coi1*-40 (see Methods).

The role of NPR1 in this response might be indirect. Thus, one scenario would be a reinforcement of the negative crosstalking between SA and MeJA. *npr1* alleles produced more SA when infected with *Pto*[26] and seemed unable to metabolize it [27]. RIM- alleles -defective in terms of SA perception- might have left intact the negative crosstalk between SA and MeJA, and an excess of SA repressed the response to MeJA beyond the wild type levels. Therefore, the RIM+ alleles would be defective in terms of both SA perception and SA-MeJA crosstalking, an explanation that would also be in agreement with the behavior of the null alleles.

To test this hypothesis, the double mutants between *npr1*-1 and *NahG* (a transgenic plant that degrades SA, [28]), *eds5* (a mutant in SA transport, [29]), and *sid2* (a mutant in SA biosynthesis, [30]), were constructed and tested for RIM. *npr1*-1 did not respond to MeJA even if the levels of SA were low (Figure 3c), so the hypothesis of a reinforcement of the negative crosstalk was not supported.

### *BOP1* and *BOP2* and their role in RIM

**Figure 4:**
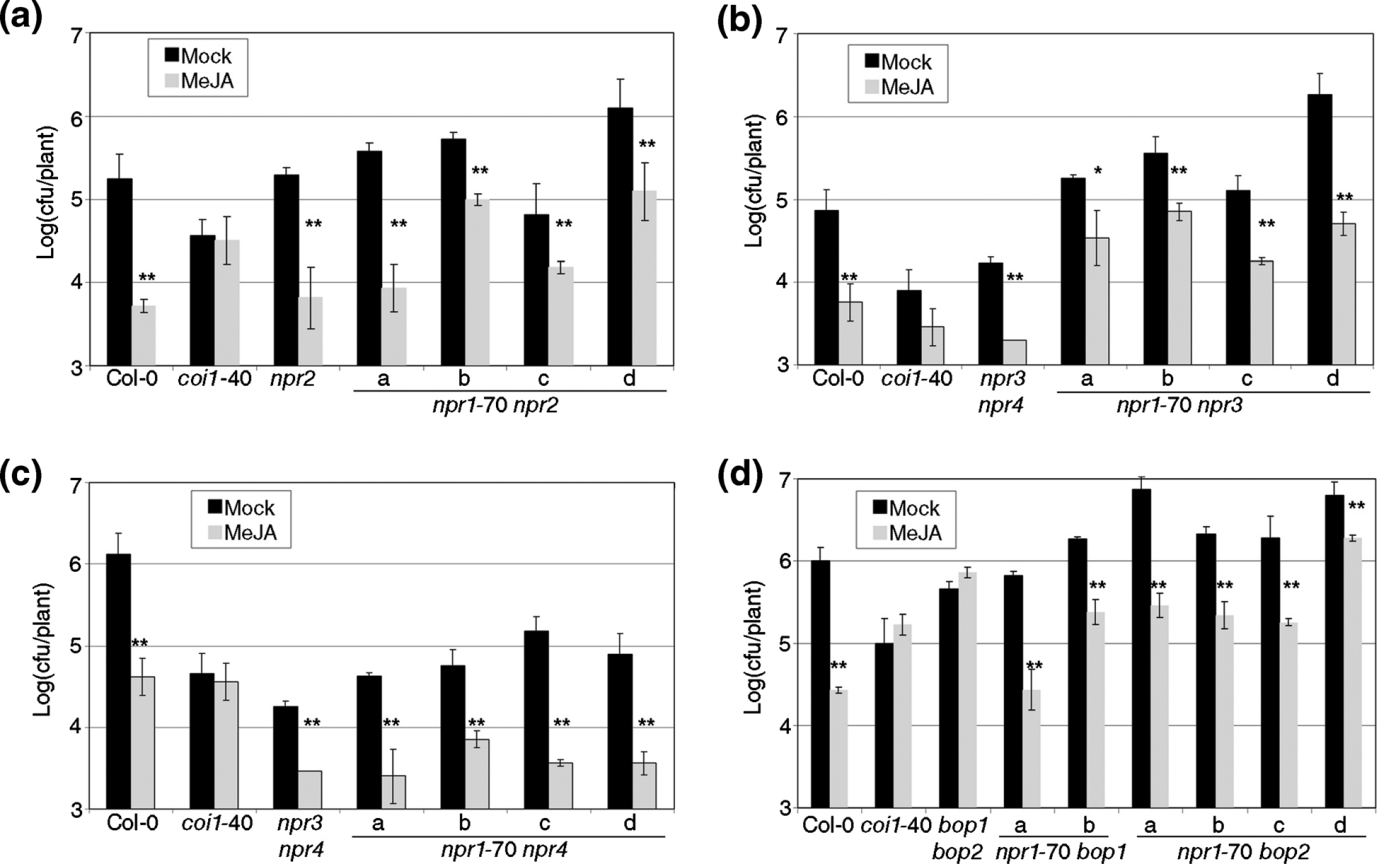
**A role for the *NPR1 *paralogs in resistance triggered by MeJA.** Double mutants of *npr1*-70 with *NPR1* paralogs and their corresponding controls were tested for resistance triggered by MeJA as described in Figure 1. **(a)** Double mutants with *npr2*. **(b)***npr3*. **(c)***npr4*. **(d)***bop1* and *bop2*. Four independent lines were tested in each case, except with *bop1*, where only two were obtained. One asterisk means a significant difference with P<0.05, and two asterisks means P<0.01 (Student’s test of one tail).

The experiments with the null alleles showed that *NPR1* was not necessary for RIM. Perhaps *NPR1* and other gene(s) would be redundantly responsible of RIM, and while null *npr1* alleles would have a RIM+ phenotype, some *npr1* alleles could be RIM- by interacting with other protein(s) negatively. The most likely candidates for these interactions were the *NPR1* paralogs, since their proteins shared the same overall structure. There are five additional paralogs of *NPR1* in the Arabidopsis genome [7], and we analyzed double mutants of *npr1*-70 with different paralogs (Figure 4). There was no proof of *NPR1* having a redundant role in RIM. *NPR2* did not play a significant role in RIM (Figure 4a) and the same was true for the double *npr3 npr4* (Figure 4b,c and [7]). Strikingly, the double *bop1 bop2*[21] was RIM- (Figure 4d).

**Figure 5:**
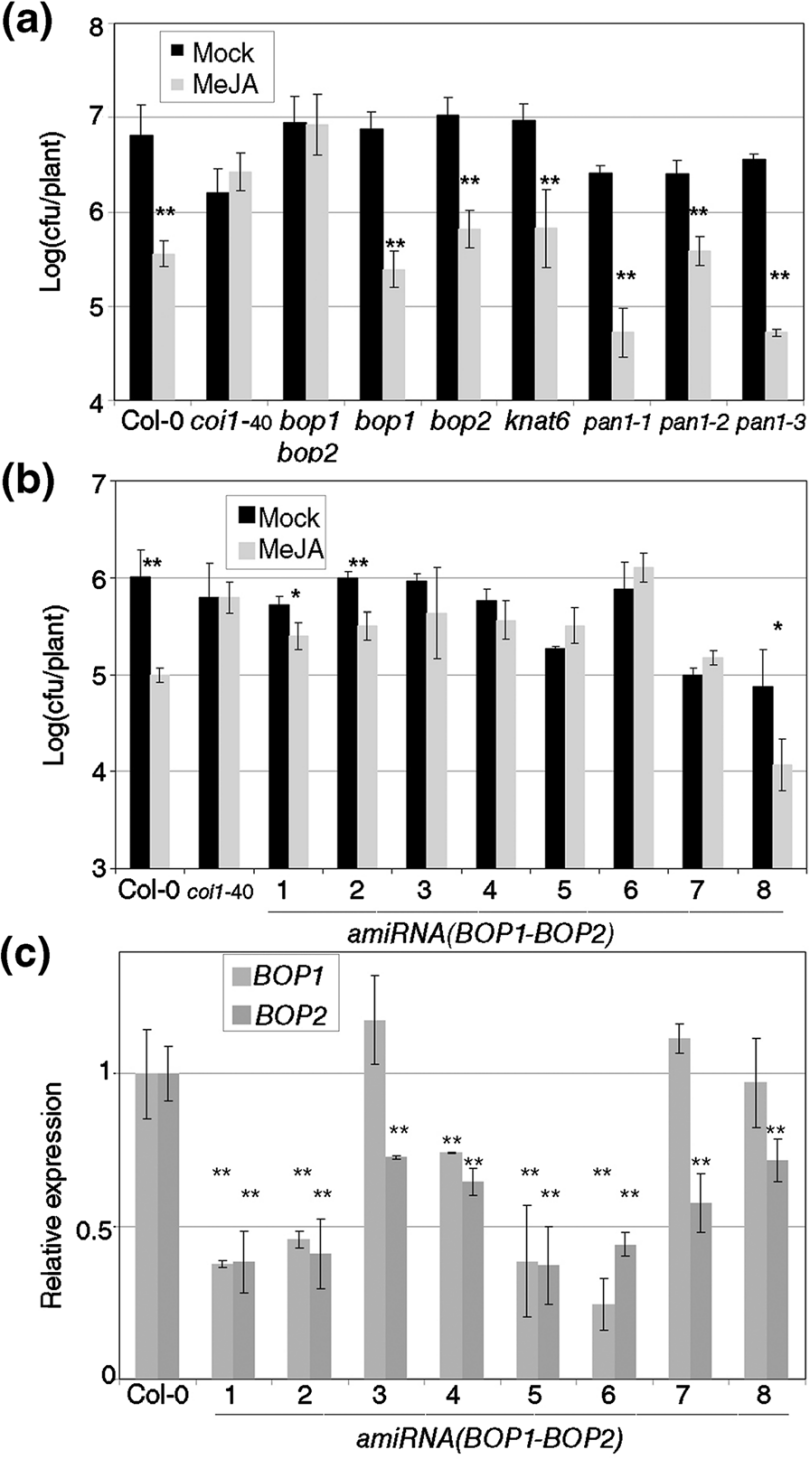
**Only the reduction of both *bop1 *and *bop2 *expression affects resistance triggered by MeJA. (a)** The double *bop1 bop2*, the single mutants, T-DNAs insertions in *KNAT6* and *PAN1*, and their controls were tested for resistance induced by MeJA as described in Figure 1. *KNAT6* and *PAN1* are genes that interact with *BOP1* and *BOP2*. **(b)** Reduction of *BOP1* and *BOP2* expression partially phenocopies the double *bop1 bop2*. Eight independent transgenic lines of an artificial micro RNA designed to reduce the levels of *BOP1* and *BOP2* (*amiRNA*(*BOP1-BOP2*) were tested as described in Figure 1. The lines did not show any macroscopic blade-on-petiole phenotype. **(c)** RNA was extracted from 3-week-old plants of the lines described in (b), and transcript levels for *BOP1* and *BOP2* were measured by means of RT-qPCR. Levels of expression are normalized to three reference genes and to the level of Col-0 in mock. Asterisks mark the significance of the difference between the levels of expression of each line with Col-0; one asterisk means a significant difference with P<0.05, and two asterisks means P<0.01 (Student’s test of one tail).

Single *bop1* and *bop2* were also tested for RIM and showed to be wild type (Figure 5a). BOP1 and BOP2 exert their function in part through transcriptional regulation of KNAT6 and physical interaction with PAN (TGA8, [31]), but T-DNA insertions predicted to disrupt KNAT6 or PAN activity did not have an effect on RIM (Figure 5a). To rule out the possibility that other mutations besides *bop1* and *bop2* were producing this RIM- phenotype, we constructed an artificial microRNA (*amiRNA*, [32]) to deplete the levels of *BOP1* and *BOP2* at the same time. Eight independent homozygous transgenic lines for *amiRNA* (*BOP1* - *BOP2*) were analyzed for RIM (Figure 5b). Five out of eight lines were RIM-, and the remaining three responded less than the wild type control. The levels of both genes were partially depleted in the eight lines (Figure 5c); five of the lines had both genes significantly reduced, and all had *BOP2* significantly reduced. None of these lines had the characteristic blade-on-petiole macroscopic phenotype, not even as the subtle phenotype of *bop1* alone.

**Figure 6:**
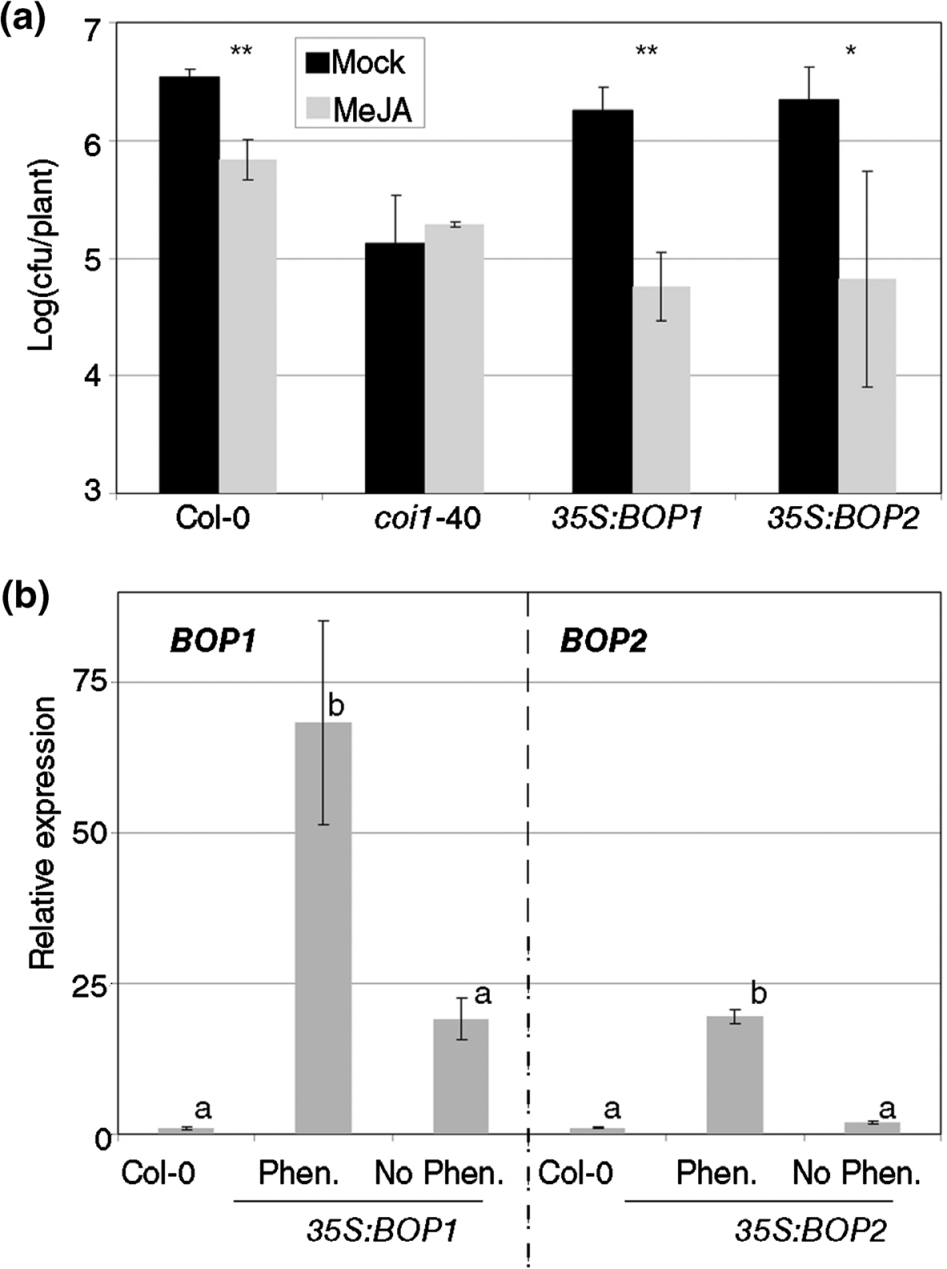
**The overexpression of *BOP1 *or *BOP2 *produces more response to MeJA. (a) ***35S:BOP1* and *35S:BOP2* lines [33] and their controls were tested as described in Figure 1. One asterisk means a significant difference with P<0.05, and two asterisks means P<0.01 (Student’s test of one tail). **(b)** Silencing of the overexpression lines. *35S:BOP1* and *35S:BOP2* lines were phenotyped at the time of bolting as having a characteristic phenotype (Phen.) [33] or being wild type (No Phen.). Then, RNA was extracted from several plants and the levels of the transgenes quantified as described in Figure 5c. On the left side of the plot, relative expression of *BOP1* and, on the right, relative expression of *BOP2*. Means with the same letter are not significantly different (Fisher's LSD test, P<0.05). The test was performed separately for each gene.

The previous experiments had shown that *BOP1* and *BOP2* were acting redundantly in RIM (Figure 4d). Therefore, increasing the amount of any of them should have an effect on RIM, especially since normal levels of *BOP1* and *BOP2* are quite low (Additional file 1, [21]). The overexpression lines of *BOP1* and *BOP2* described [33] were analyzed for RIM. *35S:BOP1* had a stronger RIM than Col-0, and *35S:BOP2* had a strong variation in the MeJA treated plants (Figure 6a). At the time of the experiments, each population looked homogeneous, but when these plants were grown to set seeds, two phenotypes could be observed in each transgenic line. Approximately half of the plants showed a wild type phenotype, and the other half reproduced the dwarf plants described [33]. Seeds from both lines and from both phenotypes reproduced the two phenotypes. It seems that it was an issue of silencing, since RNA taken from plants classified by their mentioned phenotype diverged widely in the transgene expression (Figure 6b).

### *bop1 bop2* specificity in RIM

**Figure 7:**
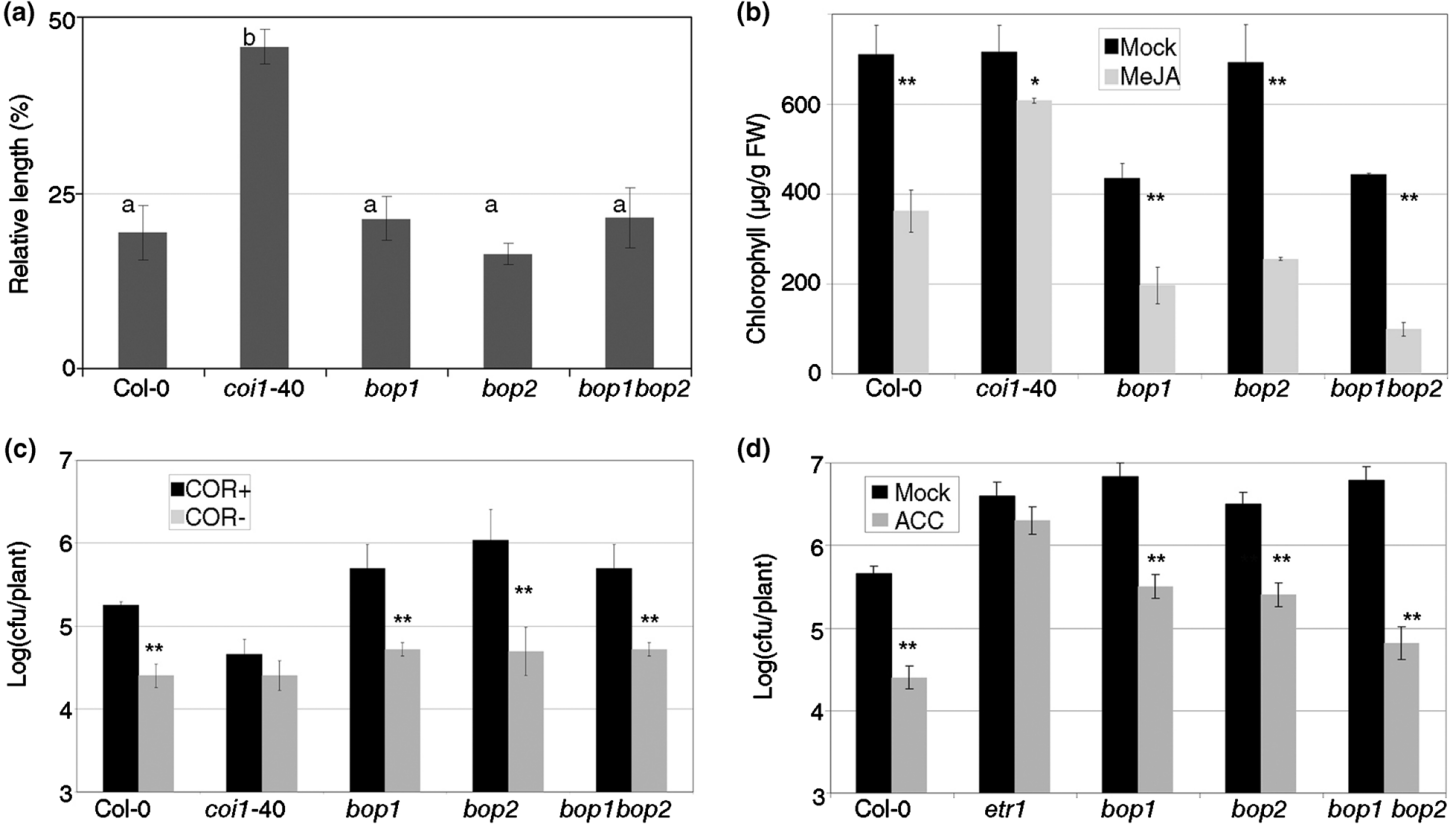
**The double *bop1 bop2 *is specifically affected in the resistance induced by MeJA. (a)** Length of primary root. *bop1 bop2* and their controls were grown in plates with Johnson's Media [58] with or without 50 μM MeJA. At the age of 10 days, the lengths of the roots were measured in both conditions and their ratio (MeJA treated divided by mock treated) expressed as percentage. Means with the same letter are not significantly different (Fisher's LSD test, P<0.05). **(b)** Senescence induced by MeJA. The indicated genotypes were grown in soil and mature leaves from 6-week-old plants were cut and floated on water with or without 100 μM MeJA. The amount of chlorophyll (in μg/g fresh weight) was measured after four days of darkness, with three groups of leaves of *c.* 1 g each. **(c)** Coronatine as a virulence factor. Bacteria with coronatine (*Pto*, COR+) or without coronatine (*Pto(cfa*^*–*^*),* COR-) were inoculated and their growth measured as in Figure 1. **(d)** Resistance induced by ethylene. The plants were treated with 1 mM 1-aminocyclopropane-1-carboxylic acid (ACC) or a mock treatment, and then *Pto* was inoculated and measured as in Figure 1. *etr1* is a negative control of resistance induced by ethylene. One asterisk means a significant difference with P<0.05, and two asterisks means P<0.01 (Student’s test of one tail).

The *bop1 bop2* double mutant lacked a RIM response, but this failure in MeJA signaling might occur at different points of the signal transduction. For example, the defect could target a general signaling component affecting all MeJA responses (e.g. *coi1,*[34]), or a specialized part of the pathway, affecting a subset of MeJA responses (e.g. *jin1,*[35]). When *bop1 bop2* plants were grown in plates containing MeJA, the growth of the roots was similar to the wild type controls (Figure 7a). Other phenotypes of *bop1 bop2* plants growing in MeJA plates were similar to the wild type controls (carotenoids production, size of aerial part, number of trichomes, etc.; data not shown). Another effect of MeJA is the increase of senescence in detached leaves, measured as chlorophyll production [36]. *bop1 bop2* responded as the wild type in this particular system (Figure 7b). Coronatine is a virulence factor of several *Pseudomonas* isolates with structural and functional similarities to JA-Ile (the functional form of Jasmonate *in planta*, [5]), therefore a mutant *Pto* that lacks coronatine [37] grows less in Col-0 than the wild type *Pto*. *bop1 bop2* was also wild type in response to *Pto* with and without coronatine (Figure 7c). Inoculations with *Plectospharaella cucumerina*, a fungus that causes more disease in MeJA mutants than in wild type plants [38], did not cause any more disease in *bop1 bop2* than in Col-0 (data not shown). If *bop1 bop2* was not a MeJA signaling mutant, but specifically in RIM, it could be defective in the other signaling required for ISR; ET. It was not; when 1 mM of 1-aminocyclopropane-1-carboxylic acid (ACC, an ET precursor) was sprayed to *bop1 bop2*, the resistance triggered was similar to that triggered in the wild type controls (Figure 7d). *etr1* (*ETHYLENE RESPONSE 1,*[39]) was included as a negative control of resistance induced by ethylene.

### NPR1 and BOPs interactions

**Figure 8:**
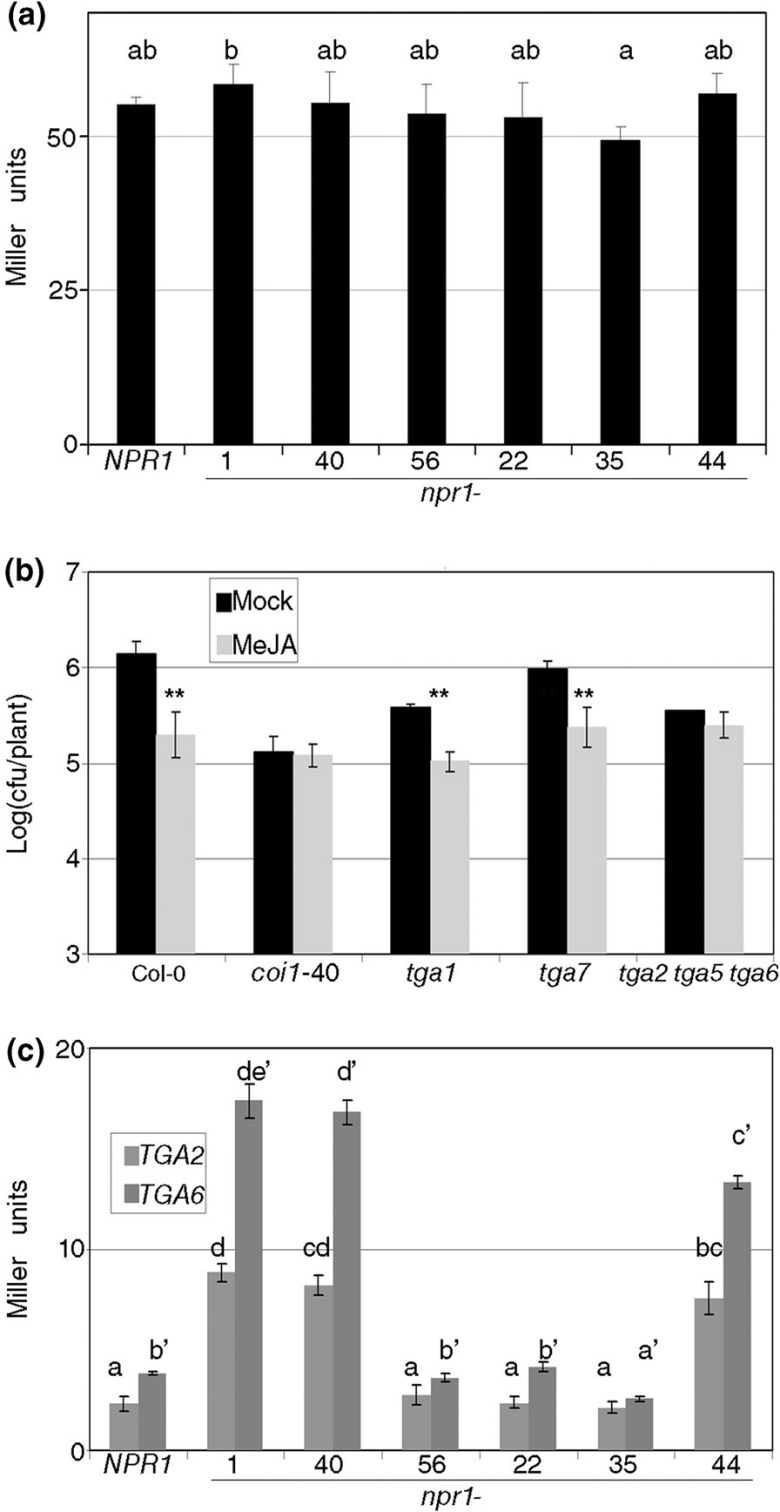
**The TGA family plays an important role in resistance induced by MeJA. (a)** Interaction of BOP1 and BOP2 in the yeast two hybrid system in presence of several *npr1* alleles cloned in a third plasmid. NPR1 is included as control, the next three alleles do not respond to MeJA in defense, and the remaining three respond as wild type. The interaction is measured in Miller Units [55]. **(b)** Null alleles of several *TGAs* alleles were analyzed as in Figure 1. *tga2,5,6* stands for the triple *tga2 tga5 tga6*. One asterisk means a significant difference with P<0.05, and two asterisks means P<0.01 (Student’s test of one tail). **(c)** Interaction between BOP1 and TGA2, and TGA6 in the yeast two hybrid system in the presence of the same *npr1* alleles of (a). Means with the same letter are not significantly different (Fisher's LSD test, P<0.05).

Once it was clear that both BOP genes are required for RIM, we tested the model that *npr1* RIM- alleles could have a dominant negative effect on BOP activity, either directly or indirectly. To first test whether NPR1 had an effect in the interaction between BOP proteins, we used a yeast two-hybrid assay. As reported, BOP1 and BOP2 interacted with each other [40]. Next, we introduced in a third plasmid containing wt *NPR1* or various mutant *npr1* alleles presented in Figure 2a. If the effect of *npr1* on RIM were a direct interaction between NPR1 and the two BOPs, the alleles that diverge in their RIM phenotype would diverge in their ability to interfere in the interaction of BOP1 and BOP2. The two classes of *npr1* alleles did not have a distinct behavior (Figure 8a, the first three *npr1* alleles are RIM-, and the last three are RIM+), therefore the dominant negative effect did not seem to be direct.

All the NPR1 paralogs tested interact with members of the TGA family in a different degree [7,21]. Therefore, the TGAs would be a reasonable candidate for being the third component, and their interaction with RIM- alleles would indirectly affect the function of BOP1 and BOP2. As a control, single mutants in *TGA1* and *TGA7* produced a significant RIM (Figure 8b), but when three specific *tgas* are knocked out at the same time (a triple which phenocopies an *npr1* mutant in SA response, [41]), there is no RIM (Figure 8b).

We reasoned that one or several of these three TGAs (TGA2, 5, and 6) might have a functional interaction with the BOPs, which might be affected by the RIM- alleles. To test this hypothesis *BOP1* and each of the mentioned *TGAs* were introduced in the yeast two-hybrid system with the *npr1* alleles mentioned above in a third plasmid. TGA2 and TGA6 interact differentially with BOP1 depending on the npr1 protein present (Figure 8c). There was an enhancement of the interaction in two out of the three RIM- alleles, and no interference in two out of the three RIM+ alleles. The interaction TGA5-BOP1 was not affected by the presence of npr1 proteins (data not shown). The experiments were repeated with BOP2 producing similar results (data not shown). In sum, the data indicated that BOPs and TGA2, TGA5 and TGA6 are required for RIM, that BOPs interact with these (and other) TGAs, and that NPR1 may modulate the affinity or stability of the interactions.

## Discussion

### NPR1 is not required for RIM

*NPR1* is an essential gene for SAR and SA perception [6]. *npr1*-1, the most widely used allele, is also impaired in RIM [13]. We speculated that since *npr1*-3 is wild type for RIM ([20,25]), and it has been reported that the difference of some phenotypes between *npr1*-1 and *npr1*-3 was due to the lack of NLS in *npr1*-3 ([17,19]), the same could be true for RIM. However, we show here that the nuclear localization of the alleles makes no difference. This conclusion is supported by multiple lines of evidence. First, the *npr1* alleles with RIM+ are not structurally similar to *npr1*-3, since not all of them are affected in the NLS (Figure 1c). Even an allele with a point mutation in the NLS (*npr1*-22, Additional file 1) should be partially localized in the nucleus [15]. Second, three RIM- and three RIM+ alleles do not differ in their nuclear localization or stability when transiently expressed in *N. benthamiana* (Figure 2a, Additional file 1). While these proteins are no longer functional, they respond to the signals of a wild type background by localizing in the nucleus. Third, when a functional NPR1 is anchored in the cytoplasm there is no complementation of the RIM- phenotype in an *npr1*-1 background (Figure 2b), nor there is an increase in RIM phenotype in an *npr1*-3 background. In fact, the application of DEX triggered an increased RIM in both backgrounds (discussed below).

But, most importantly, NPR1 is not required for RIM, since the null *npr1* alleles are RIM+ regardless of the background (Figure 3a,b). We also discarded that *NPR1* could be a part of RIM in a redundant fashion with its paralogs (Figure 4).

An interesting alternative for the role of *NPR1* in RIM would be an effect on the crosstalking between SA and MeJA. *NPR1* has been described as a key point in the negative regulation between SA and MeJA. Thus, the RIM+ alleles could be defective in both SA perception and in SA-MeJA crosstalk, while the RIM- alleles would be defective only in SA perception but not in SA-MeJA crosstalk. The inoculation with *Pto* triggers an increase in the levels of SA, and in the case of the *npr1* alleles, there is more SA than in the wild type [42]. Although this hypothesis would explain the phenotype of the null alleles, it was rejected after the experiment of Figure 3c, where a severe reduction of SA levels in a RIM- allele did not have any effect on the phenotype.

### *BOP1* and *BOP2* are redundant in RIM

The redundant functions of *BOP1* and *BOP2* are essential for normal development. Previous work has shown that the double mutant has numerous defects in plant architecture including altered leaf morphology [43], changes in floral patterning [21], defects in the conversion of shoots to flowers [44] and loss of floral-organ abscission [45]. The double mutant was tested for basal defense [21] and SA perception (Additional file 1) but no difference from wild type was found. We show here that both genes are also redundantly required in defense against pathogens triggered by MeJA. Interestingly, whereas significant loss of BOP activity is required to exert changes in development [21], RIM is abolished in plants that are only partially silenced for the BOP genes (Figure 5b,c). Thus, the levels of gene expression required for RIM are higher than those required for normal development. Compatible with this idea, *BOPs* expression in plants is highly localized, restricted to young organ primordia, leaf petioles, and lateral organ boundaries, which may make systemic responses to MeJA sensitive relatively minor changes in BOP transcript abundance. Both NPR1 and the BOPs localize to the cytoplasm as well as nucleus and interact with members of the TGA family of bZIP transcription factors, albeit with different affinities (e.g., [12]). In development, BOP1 and BOP2 form a nuclear complex with TGA8/PERIANTHIA (PAN) to regulate number of sepals and petals in flowers and potentially to promote floral meristem fate [21]. Given that *pan* loss-of-function did not reproduce the RIM- phenotype (Figure 5a) other genes, perhaps *TGAs*, are involved in this phenotype, as shown for SA perception [23]. Given that BOPs play both positive and negative roles in transcriptional regulation of the KNOX (Knotted1-like homeobox) gene *KNAT6*[46], we also tested if RIM was affected by *knat6* loss-of-function, but again, no difference was observed (Figure 5a). This may reflect redundancy with other KNOX genes, or more likely, that BOP regulation of RIM is independent of KNAT6.

Whether *bop1 bop2* recapitulates or not all the phenotypes of the RIM- *npr1* alleles (e.g. ISR, [13]; *Verticilium* resistance, [18]; resistance induced by *Piriformospora indica,*[47]; etc.) remains to be assessed. We did check that there were similar phenotypes in the specificity of response to MeJA as well as the fact that *bop1 bop2* was wild type for the rest of MeJA phenotypes (Figure 7). But there were strong differences, since *npr1*-3 is affected in basal defense and SA perception while *bop1 bop2* is wild type for both phenotypes [21]. Regarding ET, the other hormone relevant for ISR, it has been proposed that applications of this hormone could render the crosstalk between SA and MeJA independent of NPR1 [19]. It seems plausible that the ET induced resistance works as the MeJA induced resistance and other proteins -perhaps NPR1 paralogs, but not the BOPs (Figure 7d) - might also be affected by some alleles of *npr1*.

### Some *npr1* alleles interfere in the BOPs-TGAs interaction

The RIM- *npr1* alleles were the majority of the alleles found (32 RIM- vs. 11 RIM+). How is this compatible with the fact that the null *npr1* alleles are RIM+? A possible explanation was the selection used in the screening. Since the selection was made for complete loss of SA perception, perhaps most of the RIM+ alleles had a phenotype of partial SA perception, as the null alleles. Then, the prediction would be that a good number of random alleles of *npr1* would be RIM+ and partially receptive to SA. We previously showed that for SA perception, there are genetic interactions between the *npr1* alleles and the *NPR1* paralogs [11]. The work reported herein points to a genetic interaction too, this being between *npr1* alleles on one side and the *BOPs* on the other. Thus, the RIM- alleles were a phenocopy of the *bop1 bop2* mutant in defense but not in development. This discrimination was a consequence of the different thresholds for the phenotype in development and defense (Figure 5b,c).

Mechanistically, the levels of expression of the *BOPs* were low in comparison to *NPR1* (Additional file 1), so a direct or indirect negative interference of NPR1 with the BOPs would be favored stoichiometrically. Once the pathogen was inoculated, the levels of SA would rise and in a wild type plant NPR1 is degraded as part of the signaling process [48]. In an *npr1* background, this signaling would not be transmitted and perhaps the npr1 proteins would be able to interfere longer in RIM. This would explain the behavior of *NPR1-HBD* in *npr1*-1 (v 2b); NPR1-HBD in the cytoplasm did not complement *npr1*-1 in the RIM phenotype, but when DEX was applied there was complementation of the phenotype. Likely, when no DEX was present npr1-1 would somehow interfere with the function of the BOPs. When DEX was present, the presence of NPR1-HBD in the nucleus would trigger the degradation of both NPR1-HBD and npr1-1. If npr1-1 was degraded, the BOPs would function normally.

There was no evidence for a direct interaction in yeast, since the presence of NPR1 or mutated versions of this protein did not interfere in the interaction between BOP1 and BOP2 in a consistent manner with the phenotype (Figure 8a). A first alternative was that the interference of the RIM- alleles would occur with the BOPs without affecting the interaction between the BOPs. A second alternative would be that the RIM- alleles would interfere with other proteins that normally interact with the BOPs. In both cases there is a family of proteins that interacts with both NPR1 and the BOPs, the transcription factors TGAs [49], with -again- functional redundancy (Figure 8b). Two out of three RIM- alleles enhanced or stabilized the BOPs-TGAs interaction, while two out of three RIM+ alleles did not (Figure 8c and data not shown). It was clear that the npr1 mutated proteins had an unpredicted effect on the BOPs-TGAs interaction, but the yeast experiments did not produce an absolute answer about the role of npr1 proteins in RIM. We speculate that *in planta*, all the RIM- alleles enhance the interaction between the BOPs and the TGAs, titering out the TGAs and thus rendering them unable to fulfill their function of triggering defense. On the other hand, the RIM+ alleles (including the null alleles), and NPR1 would not affect the interaction either way. Since in the yeast assays two out of three alleles worked as proposed in either way, it may be possible that a factor(s) is present in the plant that is not in yeast, or perhaps the fact that there are ten *TGAs*[49], and that *NPR1* is expressed between 3 and 18 times more than *BOP1* + *BOP2* (Additional file 1) could explain this difference. If this hypothesis were to be true, it will definitively explain the role of npr1 in RIM.

## Conclusions

In sum, we have shown that, in wild type conditions, the *BOPs* and the *TGAs* (but not *NPR1*) are required for the resistance triggered by methyl jasmonate against *Pto*. We propose that the phenotype of the *npr1* RIM- alleles is caused by their interference between BOPs and TGAs.

## Methods

### Plant growth and inoculation

*Arabidopsis thaliana* (L.) Heynh. was sown and grown as described [23] in controlled environment rooms with days of 8 h at 21°C, 150 μmol m^-2^ s^-1^, and nights of 16 h at 19°C. Treatments, inoculations, and sampling started 30 minutes after the initiation of the artificial day to ensure reproducibility. The following genotypes were used: *npr1*-1 and *npr1*-3 [27]; *npr2*, *npr1*-20 to *npr1*-71, and combinations of *npr1*-70 with other genotypes [11]; *35SCaMVp:NPR1HBD*[15]; *sid2*[30]; *eds5*[29]; *NahG*[28]; *npr3* and *npr4*[7]; *bop1*-3 and *bop2*-1 [21]; *coi1*-40 (Dobón, Wulff, Canet and Tornero, to be published elsewhere); *kant6, pan1*-1 *to pan1-*3, *tga1*, and *tga7*[50]; *35S:BOP1* and *35S:BOP2*[33]; *etr1*-3 [39]; *tga2 tga5 tga6*[41]. *Pseudomonas syringae pv. tomato* DC3000 (*Pto*) was grown, inoculated and measured as described [51]. Briefly, plants of 14 days were inoculated by spray with *Pto* at OD_600_=0.1 with 0.02% Silwet L-77 (Crompton Europe Ltd, Evesham, UK). Three days later, the amount of colony forming units (cfu) per plant was quantified and represented in a logarithmic scale. When indicated, a strain of *Pto* lacking coronatine was used (*Pto(cfa*^*–*^*),*[37]). For all the experiments, at least three independent treatments were performed (three independent sets of plants sown and treated on different dates).

### Expression *in planta* and in yeast

*NPR1* and six alleles of this gene were cloned in pDONR222 or pDONR221 (Invitrogen, Barcelona, Spain) and then transferred to pMDC43 [52] for expression *in planta* with GFP and to pARC352 [53] for expression in yeast. Similarly*, BOP1, BOP2, TGA2, TGA5,* and *TGA6*, were cloned and then transferred to pDEST22 and pDEST32 (Invitrogen) for expression in yeast. Yeast n-hybrid analyses were done as described [54], and the interactions were quantified as described [55]. *N. benthamiana* leaf tissue was mounted in water under a coverslip 4 days after infiltration with *Agrobacterium tumefaciens* containing the constructs. All imaging was conducted with a Leica TCS SL confocal laser scanning microscope (Leica, Barcelona, Spain) using an HCX PL APO CS 40X/1.25 oil objective to study the subcellular localization of the fluorescence-tagged proteins. Green fluorescent protein was visualized by 488-nm excitation with an Ar laser, and its emissions were examined with a band-pass filter for 500 to 530 nm. The primers used are included as Additional file 1. Primers and chemical products were purchased from SIGMA (St. Louis, MO, USA) unless otherwise is stated. For the construction of *amiRNA(BOP1-BOP2)*, the plasmid pRS300 was modified [32], cloned in pGW14 [56], and plants were transformed as described [57].

### Chemical treatments

To measure the effect in *Pto* growth 100 μM methyl jasmonate (MeJA) in 0.1% DMSO and 0.02% Silwet L-77 (Crompton Europe Ltd) was applied by spray one day before the pathogen inoculation [25]. Dexamethasone was applied at 2 μM diluted in water from a stock of 20 mM in EtOH. 1-Aminocyclo- propane-1-carboxylic acid (ACC) was sprayed at 1 mM in water with 0.02% Silwet L-77.

### *In vitro* growth

For *in vitro* culture, plants were grown in Johnson’s media [58] with 1 mM KH_2_PO_4_. When indicated, the plates were supplemented with 50 μM MeJA. The length of the roots was measured with ImageJ software [59]. Senescence induced by MeJA was measured as described [36].

### RT-qPCR

Total RNA from 3-week-old (Figure 5c) or 6-week-old plants (Figure 6b) was extracted with Trizol (Invitrogen), following the manufacturer’s instructions. cDNA was synthesized with RevertAid™ First Strand cDNA Synthesis Kit (Fermentas, Madrid, Spain), and the quantitative PCR performed with LuminoCt Sybr Green qPCR Ready Mix (SIGMA) in a 7000 RT-PCR Systems machine (Applied Biosystems, Madrid, Spain), following the manufacturer’s instructions. For each measurement three biological replicates were done. The obtained values were referred to the geometric average of three reference genes (At3G18780, At1G49240, and At5G60390), as described [60], and normalized, being the value of Col-0 in mock equal to one. The list of primers used is provided in Additional file 1.

## Competing interests

The authors declare that they do not have any competing interest.

## Authors' contributions

JVC, AD, and JF performed the experiments, analyzed the data and revised the paper. PT designed the research, analyzed the data and wrote the paper. All authors read and approved the final manuscript.

## Supplementary Material

Additional file 1 **Figure S1.** Localization of cloned *npr1* alleles mentioned in the text. **Figure S2.** NPR1HBD treated with DEX is more sensitive to BTH. **Figure S3.** Expression levels of NPR1 paralogs. **Figure S4.** Response of *bop1 bop2* to SA and BTH. **Table S1.** List of primers used. Click here for file
