# Supplementary material for: The BLADE-ON-PETIOLE genes of Arabidopsis are essential for resistance induced by methyl jasmonate
Source: BMC Plant Biol. 2012 Nov 2;12:199. doi: 10.1186/1471-2229-12-199 (PMC3570372; doi:10.1186/1471-2229-12-199)
Supplement: Additional file 1 — Figure S1. Localization of cloned npr1 alleles mentioned in the text. Figure S2. NPR1HBD treated with DEX is more sensitive to BTH. Figure S3. Expression levels of NPR1 paralogs. Figure S4. Response of bop1 bop2 to SA and BTH. Table S1. List of primers used. [file 1471-2229-12-199-S1.pdf]

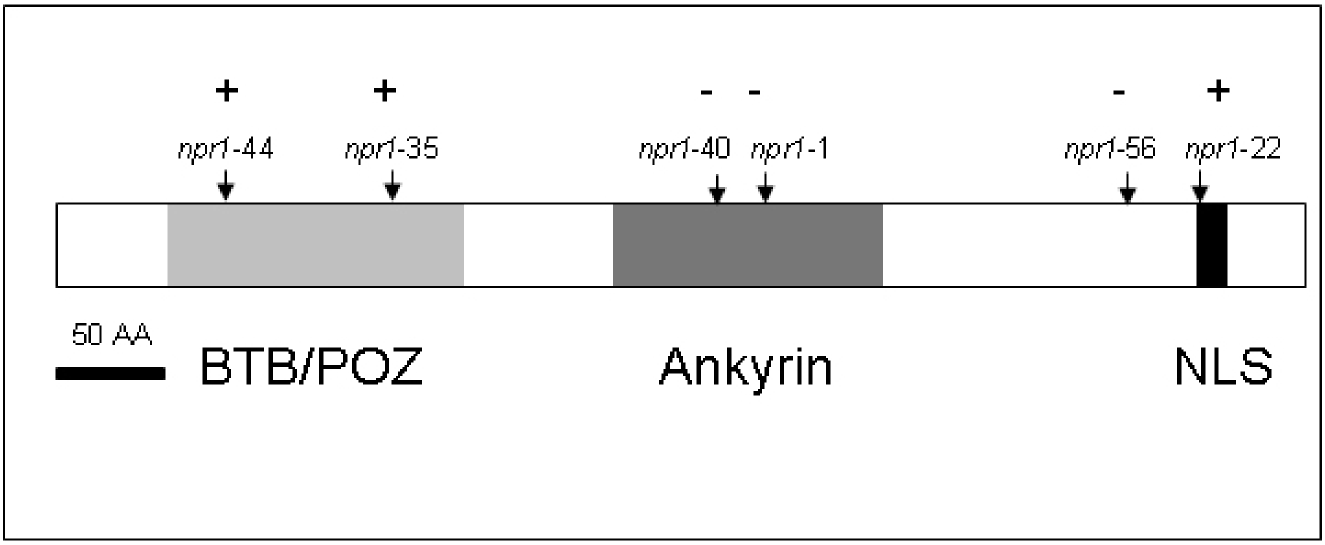

Supplemental Fig. 1. Localization of cloned *npr1* alleles mentioned in the text. These alleles are used in Fig 2, 8. As in Fig. 1C, BTB/POZ stands for Broad-Complex, Tramtrack and Bric-a-brac proteins, Pox virus and Zinc finger proteins. Ankyrin for Ankyrin Repeat Motifs (4 of them) and NLS for Nuclear Localization Signal. The arrows indicate point mutations. The plus sign indicates a RIM wild type response, while the minus sign indicates a lack of RIM.

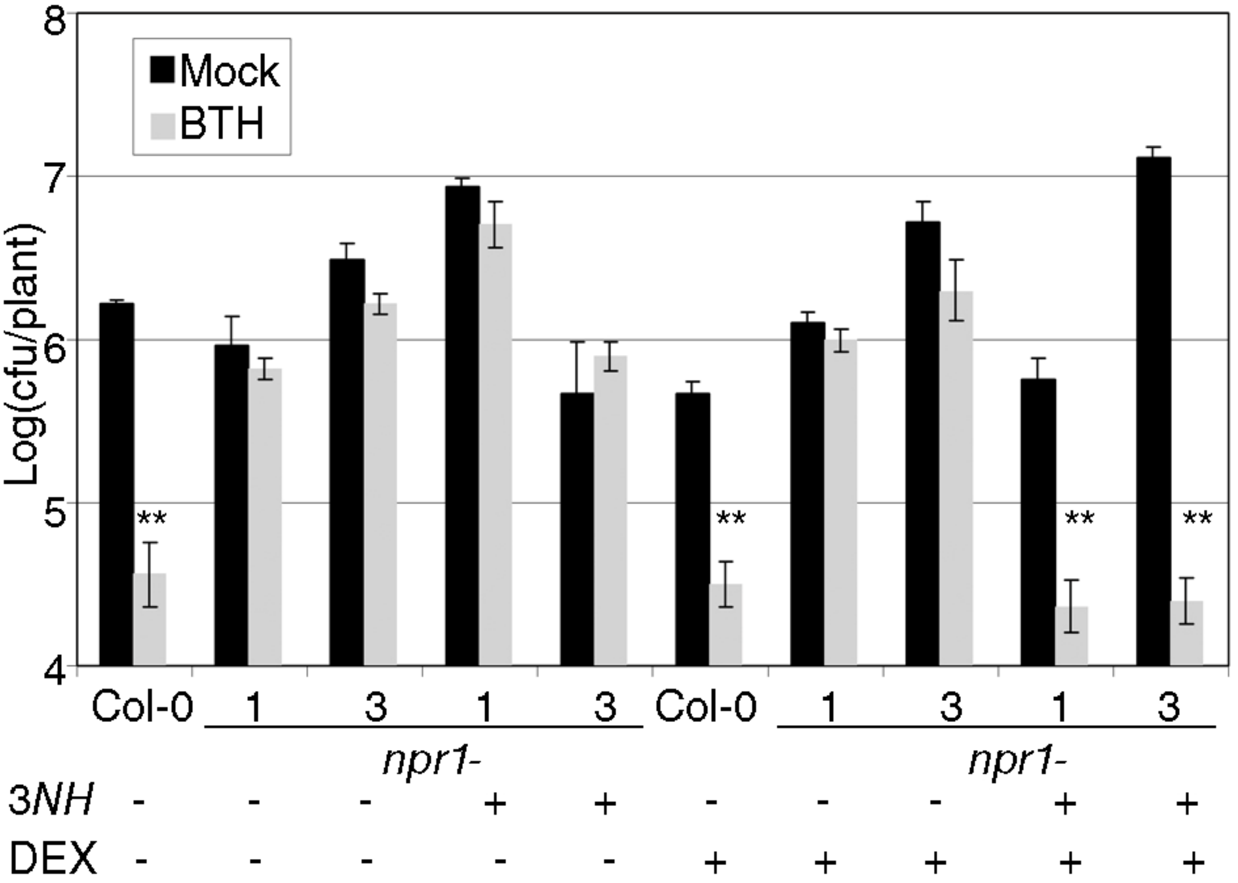

Supplemental Fig. 2. NPR1HBD treated with DEX is more sensitive to BTH. As a control, the same lines described in Fig. 2B were treated with and without 350 µM BTH, and with and without DEX. BTH (Benzothiadiazole, in the form Bion® 50 WG, a gift from Syngenta, Madrid, Spain) is an analogue of SA that triggers a strong resistance in plants with a functional NPR1 protein. DEX stands for dexamethasone and *3NH* for *35S:NPR1-HBD.*

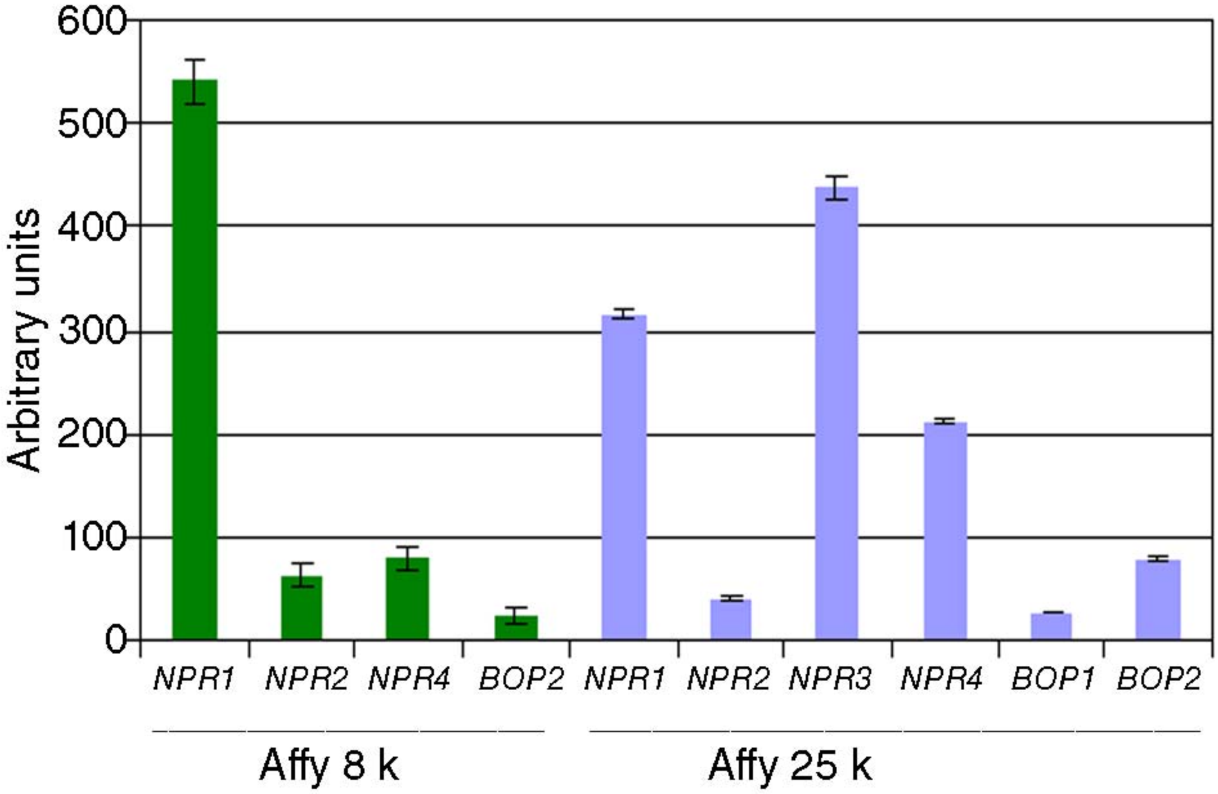

Supplemental Fig. 3. Expression levels of NPR1 paralogs. The levels of expression of *NPR1* and its paralogs were obtained from TAIR (www.arabidopsis.org) for the Affymetrix AG 8 k microarray (green) and for the Affymetrix ATH1 25 k microarray (blue).

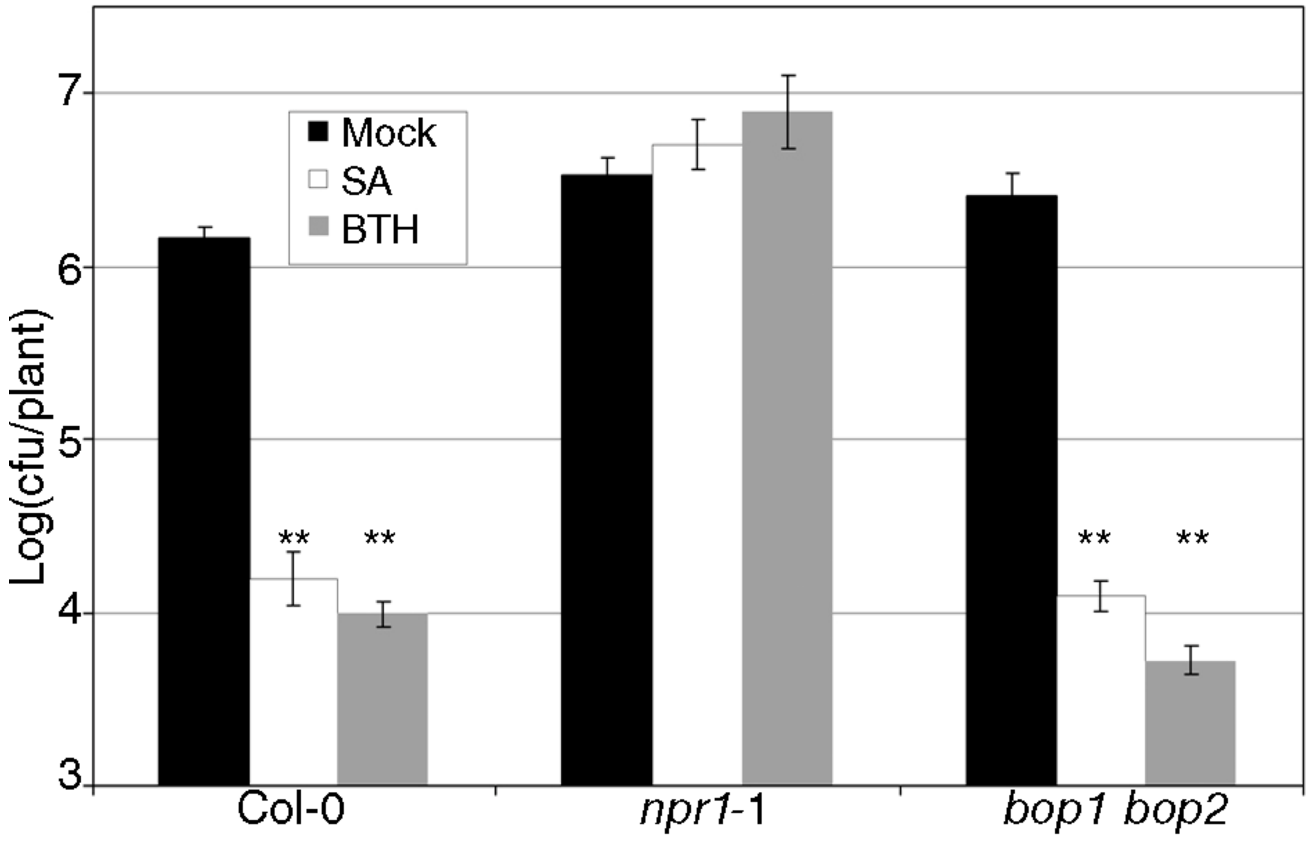

Supplemental Fig. 4. *bop1 bop2* is able to perceive SA and BTH. The response of the double mutant *bop1 bop2* to SA and BTH was tested, along Col-0 and *npr1*-1. SA was applied as 500 µM, and BTH as 350 µM, one day prior to *Pto* inoculation.

Supplemental Table 1 List of primers used.

Name	Sequence	Description
NPR1GWF2	GGGGACAAGTTTGTACAAAAAAGCAGGCTT CATGGACACCACCATTGATGG	Cloning NPR1 in pDONR222
NPR1GWR2	GGGGACCACTTTGTACAAGAAAGCTGGGTT TTACCGACGACGATGAGAGAGT	Cloning NPR1 in pDONR222
TGA2GWF2	GGGGACAAGTTTGTACAAAAAAGCAGGCTT CATGGCTGATACCAGTCCGAG	Cloning TGA2 in pDONR221
TGA2GWR2	GGGGACCACTTTGTACAAGAAAGCTGGGTT TTACTCTCTGGGTCGAGCAAGCC	Cloning TGA2 in pDONR221
TGA5GWF	GGGGACAAGTTTGTACAAAAAAGCAGGCTT CATGGGAGATACTAGTCCAAGAAC	Cloning TGA5in pDONR222
TGA5GWR	GGGGACCACTTTGTACAAGAAAGCTGGGTT TCACTCTCTTGGTCTGGCAAGCCATAG	Cloning TGA5 in pDONR222
TGA6GWF	GGGGACAAGTTTGTACAAAAAAGCAGGCTT CATGGCTGATACCAGTTCAAGGAC	Cloning TGA6 in pDONR222
TGA6GWR	GGGGACCACTTTGTACAAGAAAGCTGGGTT TCACTCTCTTGGCCGGGCAAGCCACAAG	Cloning TGA6 in pDONR222
BOP1GWF	GGGGACAAGTTTGTACAAAAAAGCAGGCTT CATGAGCAATACTTTCGAAGA	Cloning BOP1 in pDONR222
BOP1GWR	GGGGACCACTTTGTACAAGAAAGCTGGGTT CTAGAAATGGTGGTGGTGGTGA	Cloning BOP1 in pDONR222
BOP2GWF	GGGGACAAGTTTGTACAAAAAAGCAGGCTT CATGAGCAATCTTGAAGAATC	Cloning BOP2 in pDONR222
BOP2GWR	GGGGACCACTTTGTACAAGAAAGCTGGGTT CTAGAAGTGATGTTGATGATGG	Cloning BOP2 in pDONR222
3rd_set_I	GATTTCAATGTGAGTCTATCCTGTCTCTCTTTTGTATTCC	amiRNA(BOP1+BOP2)
3rd_set_II	GACAGGATAGACTCACATTGAAATCAAAGAGAATCAATGA	amiRNA(BOP1+BOP2)
3rd_set_III	GACAAGATAGACTCAGATTGAATTCACAGGTCGTGATATG	amiRNA(BOP1+BOP2)

3rd_set_IV	GAATTCAATCTGAGTCTATCTTGTCTACATATATATATTCCT	amiRNA(BOP1+BOP2)
BOP1qF	GCTCGCTACTTTGGCGTCG	RT-qPCR BOP1
BOP1qR	CTTCAATGGAGGCTTTCTCCAC	RT-qPCR BOP1
BOP2qF	GTTACTTCGGCGTCGAGCAG	RT-qPCR BOP2
BOP2qR	CATCTTCGATAGAGGCTTTCTCCA	RT-qPCR BOP2
